# Strain Agnostic Influenza Virus Propagation in a Serum‐Free, Suspension‐Adapted MDCK Cell Line

**DOI:** 10.1111/irv.70237

**Published:** 2026-03-17

**Authors:** Jessica B. Huskey, Michelle L. Rock, Pooja V. Chaudhary, Emily C. Hill, Madeline E. Hoover, Nicole M. Rideout, Kamerin D. Dean, Thomas Scott Alderman, Phong Ho, M. Anthony Moody, Gregory D. Sempowski, Thomas H. Oguin

**Affiliations:** ^1^ Duke Human Vaccine Institute, Duke University School of Medicine Durham North Carolina USA; ^2^ RTI International Research Triangle Park North Carolina USA

**Keywords:** influenza A virus, influenza B virus, suspension culture, viral propagation

## Abstract

**Background:**

The continuing circulation and evolution of seasonal influenza viruses remains a public health and socioeconomic threat on a global scale. Viral surveillance and vaccination of the public have been relied upon to confer and boost immunity in the population. Traditionally, influenza strains are propagated in embryonated chicken eggs, but this process remains imperfect and subject to genetic drift of the virus and a reliable source of eggs. Cell culture‐based propagation of influenza virus has recently been commercialized, but this method has been difficult to adapt to lab settings.

**Methods:**

Madin‐Darby canine kidney (MDCK) cells were adapted to thrive in serum‐free growth in suspension. The suspension MDCK (sMDCK) line was characterized by measuring replication and viability during routine passage and infection. Fifteen different influenza strains were propagated using this model and were assayed to determine hemagglutination and plaque forming units and compared to influenza strains grown in adherent cell culture. Microneutralization tests were also conducted to ensure each strain maintained the proper antigenicity.

**Results:**

The cell line was successfully adapted to serum‐free growth in suspension. For each virus strain, the sMDCK platform successfully produced a virus stock in 1–3 days. Additionally, sMDCK progeny virus maintained its antigenicity based on neutralization assays.

**Conclusions:**

This simple, scalable method was used to reliably propagate 15 influenza strains with the elimination of costly reagents and animal serum. The results are comparable to traditional methods, and the protocol presented in this work could be adapted to nearly any laboratory setting.

## 1 Introduction

Influenza virus infections remain a global agricultural, socioeconomic, and public health threat. Every year, substantial efforts are made to conduct viral surveillance, determine vaccine strain recommendations, and produce seasonal vaccine formulations for global distribution. Influenza virus infects 3%–16% of the United States population annually (CDC, Flu Burden), resulting in mild to severe respiratory infection [1, 2, 3], and annual global mortality following influenza virus infection ranges from nearly 300,000 to 650,000 [4]. As such, the virus poses a significant public health risk and pronounced economic burden, amounting to an estimated cost of $47.2–$149.5 billion in the United States alone [1, 5]. Currently there are two subtypes of seasonal Influenza A circulating in the human population and causing disease, H1N1 and H3N2, and until recently, two Influenza B lineages, Victoria and Yamagata [6, 7]. As seasonal vaccination is the best strategy to reduce disease burden and the spread of the virus, it is essential to produce quality viral products for research in diagnostics, epidemiology, and vaccine development.

Currently, most commercial influenza virus propagation is performed in embryonated chicken eggs. There are numerous benefits to this method, namely that the protocol is incredibly reliable and can be used to successfully propagate a wide range of influenza strains. Despite its universal acceptance as a method of influenza virus propagation, it has multiple drawbacks [8]. This approach requires millions of eggs and remains costly and vulnerable to changes in egg supply. There are also concerns about the scalability and responsiveness in the event of a pandemic using egg‐based systems, especially if the outbreak strain were of avian origin [9]. The resulting viral products can be difficult to purify, subject to antigenic alteration as a result of egg adaptation, and contain potential allergens [1, 10, 11].

In the last decade, adherent cell line‐based propagation has presented a promising alternative for producing influenza vaccine strains, although it has not yet supplanted egg‐based propagation. At this time, Seqirus CSL is the only FDA‐approved cell‐based vaccine manufacturer utilizing Madin‐Darby canine kidney (MDCK) cells, which have proven widely susceptible to both influenza A and B vaccine strains. Furthermore, cell culture propagation techniques offer more scalability, flexibility, and can avoid the potential allergens associated with egg propagation [11]. However, adherent cell culture often requires animal products such as fetal bovine serum (FBS), an expensive reagent with poorly defined composition that varies from lot to lot [12]. Furthermore, the presence of animal serum in vaccine products carries many risks, as it could be contaminated with mycoplasma, viruses, bacteria, or prions [13]. A serum‐free influenza propagation method in suspension cell culture would afford all of the benefits of adherent methods, while increasing scalability, decreasing cost, and eliminating the potential drawbacks of using animal serum [1].

Suspension cell lines may be more amenable to large scale production in bioreactors due to a greater cell surface area being exposed to media, facilitating cell growth and the removal of metabolic byproducts [14]. Compared with adherent cells, suspension cell cultures may reach higher densities, potentially improving overall yield of propagated virus. Adapting an adherent cell line to suspension culture can be achieved by serum reduction or the introduction of the *siat7e* gene [15], but as noted above, elimination of animal serum from virus propagation brings additional benefits. Thus, a readily accessible suspension cell line for influenza vaccine production could facilitate the development of new vaccine candidates and provide a more straightforward path to large‐scale production.

While numerous reports have demonstrated the propagation of influenza A strains [1, 9, 11, 15, 16, 17, 18, 19, 20], there is less publicly available data on the propagation of influenza B strains. Having a unified propagation method for multiple components of a multivalent influenza vaccine could also facilitate the development of future vaccine candidates.

In this report, we describe the simultaneous adaptation of commercially available adherent MDCK (ATCC, CCL‐34) cells to suspension and serum‐free culture, as well as the subsequent subculturing practices to produce a robust population of suspension MDCK (sMDCK) cells. To validate the utility of this protocol, the sMDCK cell line was used to propagate five H1N1, five H3N2, and five influenza B strains, including both the Victoria and Yamagata lineages. Throughout the propagation, cell viability was monitored, and supernatant samples were collected at 24‐h intervals over the course of 3 days. The viral titer of each sample was then quantified using plaque and hemagglutination assays. To ensure progeny viruses maintain their parental antigenicity, viruses produced using the sMDCK platform were also tested against proven antisera for neutralization. In comparison to similar approaches using adherent cell lines, this method is serum‐free and has been used to successfully produce up to a liter of influenza virus with standard cell culture shaker flasks in standard‐sized incubators. Additionally, the sMDCK platform is compatible with the propagation of endemic flu strains that have previously circulated in the human population. In this work, we offer a useful alternative to traditional egg or adherent cell‐based viral propagation that is inexpensive, simple, and compatible with a wide range of influenza strains.

## 2 Materials and Methods

### 2.1 Suspension Adaptation and Culturing Conditions

Adherent MDCK cells were thawed and cultured for eight passages in 1X Minimum Essential Medium (MEM) (Gibco, 11095‐080) containing 10% v/v FBS (Gibco, 10091‐148) in T‐75 flasks (Corning, 430641U) at 37°C, 5% CO_2_. Cells were then directly adapted to grow in suspension using serum‐free 4Cell MDCK medium (Sartorius, CFV3FA2003) supplemented with 8 mM L‐glutamine (Gibco, 25030) for nine passages. The suspension cells were subcultured every 3–4 days at 37°C, 5% CO_2_, 100 rpm in shaker flasks (Corning, 431143). After completing adaptation of the MDCK cells, a research cell bank (RCB) was generated after five additional passages, and 1 mL aliquots (growth medium with 5% dimethyl sulfoxide [DMSO]) were frozen in cryovials for later use.

To expand cells for viral propagation, aliquots of sMDCK cells were thawed and suspended at a concentration of 5 × 10^5^ cells/mL in Sartorius 4Cell MDXK Medium (1010‐0001) supplemented with 8 mM L‐glutamine and 1% Penicillin–Streptomycin (Gibco, 15140) and shaken at 120 rpm at 37°C, 5% CO_2_. Two days after thawing, cells were spun down at 330 × g, 4°C for 5 min and resuspended in fresh growth medium to 5 × 10^5^ cells/mL. Cell viability was then monitored every 2 days by trypan blue exclusion assay using a Countess II Automated Cell Counter (Life Technologies, AMQAX1000). Every fourth day, cells were split back to a density of 5 × 10^5^ cells/mL in fresh growth medium and returned to the incubator.

### 2.2 sMDCK Infection for Virus Propagation

For influenza virus propagation, the optimal seeding density of sMDCK cells was 5 × 10^5^ cells/mL in an infection medium containing Sartorius 4Cell MDXK Medium supplemented with 8 mM L‐glutamine, 1% Penicillin–Streptomycin, and 3 μg/mL L‐(tosylamido‐2‐phenyl ethyl) chloromethyl ketone (TPCK)‐treated trypsin (Sigma, T1426), which was suspended in water supplemented with 20 mM CaCl_2_ and 1 mM HCl. The viral propagations were completed with an MOI of 0.001 and 0.01 for the influenza A and influenza B viruses, respectively. To infect, influenza virus was diluted to the appropriate MOI in 10% of the final desired propagation volume. Cells were pelleted by centrifugation at 330 × g, 4°C for 5 min and resuspended in the diluted virus. This mixture was returned to the incubator and shaken for 1 h at 120 rpm at 37°C, 5% CO_2_. After the initial infection step, the volume was increased to the final volume with infection medium and incubated for up to 72 h at 120 rpm, 37°C, 5% CO_2_. Three replicate flasks were infected for each influenza strain, as well as an uninfected control flask; representative data are shown for the uninfected controls. Small volumes of supernatant were collected from each flask at time of infection, as well as 24, 48, and 72 h after infection. These samples were frozen at −80°C and archived for use in plaque and hemagglutination assays. The cell viability at each of these time points was determined by trypan blue exclusion assay using a Countess II Automated Cell Counter with percent viability used as a correlate of cytopathic effect formation.

### 2.3 Adherent MDCK Infection for Virus Propagation

Confluent T‐175 flasks of adherent MDCK cells were infected with influenza virus at the same MOIs used for sMDCK propagations (MOI 0.001 for Influenza A and MOI 0.01 for Influenza B). Influenza virus was diluted in 5 mL infection medium containing 1X MEM, 0.12% Bovine Serum Albumin (BSA) (Gibco, 15260‐037), 1% penicillin–streptomycin, 1X MEM nonessential amino acids (NEAAs) (Gibco, 11140‐050), and 1 mM sodium pyruvate (Gibco, 11360‐070), 10 mM (2‐[4‐(2‐hydroxyethyl)piperazin‐1‐yl]ethanesulfonic acid) (HEPES) buffer solution (Gibco, 15630‐080). Growth medium was removed from propagation flasks and flasks were washed twice with 1X phosphate buffered saline (PBS) pH 7.4 (Gibco, 20012) before diluted virus was added. Flasks were incubated at 37°C, 5% CO_2_ for 1 h with tilting every 10–15 min. After the incubation, 25 mL of infection medium was added to each flask and flasks were returned to the incubator. Three replicate flasks were infected for each influenza strain, as well as an uninfected control flask. At the time of infection, small volumes of supernatant were collected from each flask and stored at −80°C. Sample collection was repeated at 24, 48, and 72 h after infection. These samples were later used to measure virus titer at each time point in plaque and hemagglutination assays.

### 2.4 Viruses and Antisera

A list of viruses and antisera used can be found in Tables 1 and 2, respectively.

**TABLE 1:** Infectious influenza viruses were acquired from the following sources.

Virus	Subtype/lineage	Vendor	Catalog
A/Brisbane/59/2007 *	H1N1	BEI Resources	NR‐12282
A/Puerto Rico/8/1934		Charles River	10100374
A/Solomon Islands/3/2006 *		IRR	FR‐331
A/California/07/2009 *		IRR	FR‐458
A/Michigan/45/2015 *		IRR	FR‐1483
A/Texas/71/2017	H3N2	IRR	FR‐1622
A/Aichi/2/1968		BEI Resources	NR‐3177
A/Hong Kong/1/1968		BEI Resources	NR‐28620
A/Texas/50/2012 *		IRR	FR‐1210
A/Singapore/INFIMH‐16‐0019/2016 *		IRR	FR‐1590
B/Colorado/6/2017 *	Victoria	IRR	FR‐1592
B/New York/PV01181/2018		Florian Krammer laboratory	
B/Florida/04/2006 *	Yamagata	IRR	FR‐15
B/Massachusetts/2/2012 *		IRR	FR‐1196
B/Phuket/3073/2013 *		Ted Ross laboratory	

* Indicates virus strains that have been included in previous seasonal influenza vaccine formulations.

**TABLE 2:** Antisera and normal sera for antigenicity studies were acquired from the following sources.

Sera name	Vendor	Catalog
Mouse anti‐A/Brisbane/59/2007 (H1N1)	IRR	FR‐283
Mouse anti‐A/Puerto Rico/8/1934 (H1N1)	DHVI animal models unit	
Ferret anti‐A/Solomon Islands/3/2006 (H1N1)	BEI Resources	NR‐19262
Ferret anti‐A/California/07/2009 (H1N1)	IRR	FR‐359
Goat anti‐Influenza A (H1N1) pdm09	IRR	FR‐1561
Ferret anti‐A/Texas/71/2017 (H3N2)	DHVI animal models unit	
Guinea pig anti‐A/Aichi/2/1968 (H3N2)	BEI Resources	NR‐3126
Goat anti‐A/Hong Kong/1/1968 (H3N2)	BEI Resources	NR‐3118
Ferret anti‐A/Texas/50/2012 (H3N2)	IRR	FR‐1263
Ferret anti‐A/Singapore/INFIMH‐16‐0019/2016 (H3N2)	IRR	FR‐1618
Ferret anti‐B/Florida/04/2006 (Yam.)	IRR	FR‐391
Ferret anti‐B/Massachusetts/2/2012 (Yam.)	IRR	FR‐1265
Goat anti‐B/Victoria lineage	IRR	FR‐1613
Goat anti‐B/Yamagata lineage	IRR	FR‐1409
Normal mouse serum	Sigma‐Aldrich	M5905‐10ML
Normal ferret serum	DHVI animal models unit	
Normal goat serum	Gibco	16210‐064
Normal guinea pig serum	Abcam	ab7482

### 2.5 Hemagglutination Assay

Turkey red blood cells (RBCs) in Alsever's solution, supplied by Lampire Biological Laboratories (7249408), were washed three times with cold 1X PBS. The RBCs were then resuspended in cold 1X PBS and counted using a Countess II Automated Cell Counter and adjusted to a density of 1.0 × 10^7^ cells/mL. Samples were assayed in duplicate in 96‐well microtiter U‐bottom plates. To dilute, 50 μL of PBS supplemented with 0.05% BSA was added to each well, and virus was added in equal volume to the first well in the dilution series. Samples were serially diluted 2‐fold, and RBC controls not containing virus were included on every plate. After diluting, 50 μL of RBC solution was added to each well, and plates were incubated at room temperature for 20–40 min, then tilted at an angle on a light board for 5–15 min. To determine viral titers, plates were scored for the presence of hemagglutination compared to the noninfected RBC control. Hemagglutination unit (HAU) titer for each dilution was recorded as the reciprocal of the last dilution showing complete agglutination of the RBCs. For each sample the HAU value of the duplicates was averaged by geometric mean.

### 2.6 Plaque Assay

Viral titers were determined by plaque assay using adherent MDCK cells in 12‐well plates. Cells were seeded at a density of approximately 1.5 × 10^5^ cells/well in growth medium containing 1X MEM, 1% penicillin–streptomycin, 1X MEM NEAA, 10% heat‐inactivated FBS (GeminiBio, 100‐106), and 1 mM sodium pyruvate, and incubated overnight at 37°C, 5% CO_2_.

Samples were diluted tenfold from 10^−1^ to 10^−6^ in infection medium containing 1X MEM, 0.12% BSA, 1% penicillin–streptomycin, 1X MEM NEAA, and 1 mM sodium pyruvate, 10 mM HEPES buffer solution. Growth medium was removed from 12‐well plates and cells were washed with 1X PBS. To infect, PBS was removed and 200 μL of diluted sample was added to duplicate wells. Plates were incubated for 1 h at 37°C, 5% CO_2_ with periodic tilting every 10–15 min. After incubation, viral suspensions were left in the wells and 1 mL of overlay containing 1X DMEM (Gibco, 12800‐017), 0.01% diethylaminoethyl (DEAE)‐dextran (Sigma, D9885), 0.375% sodium bicarbonate (Gibco, 25080‐094), 1% penicillin–streptomycin, 0.1% BSA, 1.2% Avicel (FMC, RC‐581), and 1–3 μg/mL of TPCK‐treated trypsin was added to each well. Assay plates were incubated at 37°C, 5% CO_2_ for 3 days. On day three, wells were flooded with 10% neutral buffered formalin. After fixing, plates were washed in tap water and wells were stained with 0.1% crystal violet w/v in water for at least 5 min. After washing off excess crystal violet, plaques were counted and plaque forming unit (PFU) per mL titers were calculated and replicates averaged for each sample.

### 2.7 Influenza Virus 50% Tissue Culture Infectious Dose Assay

In order to calculate virus input for microneutralization assays, virus titers were determined using a 50% tissue culture infectious dose (TCID_50_) assay as described in the World Health Organization's Serological Diagnosis of Influenza by Microneutralization Assay [21]. Briefly, virus stocks were diluted 1:10 serially in infection medium containing 1X DMEM (Gibco, 11965‐092), 1% BSA, 1% penicillin–streptomycin, 25 mM HEPES buffer solution, and 2–3 μg/mL TPCK‐treated trypsin to produce four starting dilutions. Each starting dilution was diluted using half‐log serial dilutions in duplicate in a clear, flat‐bottom 96 well plate, leaving only infection medium in the last column as an uninfected cell control. MDCK cells (London strain; IRR, FR‐58) were added to diluted virus at 1.5 × 10^4^ cells/well. Plates were then incubated at 37°C, 5% CO_2_ overnight. After incubation, medium was removed from plates and cells were fixed with 10% neutral‐buffered formalin. A cell‐based ELISA was used to detect the presence of intracellular virus. For influenza A, biotinylated A1 clone (Millipore Sigma, MAB8257B) and A3 clone (Millipore Sigma, MAB8258B) primary antibodies were used, followed by a horseradish peroxidase (HRP)‐conjugated streptavidin (BD Biosciences, 554066) secondary reagent. For influenza B, biotinylated anti‐Influenza B clone 22D5‐12‐13 (Millipore Sigma, MAB8671B) was used as the primary antibody and was detected with the HRP‐conjugated streptavidin reagent. The HRP substrate used was *o*‐phenylenediamine dihydrochloride (Sigma, P8287) at 5 mg/mL prepared in 0.05 M phosphate–citrate buffer (Sigma, P4922). The reaction was stopped with 0.5 N sulfuric acid (Sigma, 320501). Optical density (OD) was measured at 490 nm with a Synergy H1 microplate reader (BioTek Instruments, Winooski, VT). During analysis, samples were counted as positive for virus if the signal was greater than two times the average of the uninfected cell control, and the average TCID_50_ of the virus was calculated using the Reed–Muench method [22].

### 2.8 Influenza Virus Microneutralization Assay

Microneutralization assays were performed as described [23]. Antiserum specific to each virus was used as a positive control, while a species‐matched naïve serum was used as a negative control. The sera were diluted to a starting concentration of 1:20 in infection medium containing 2–3 μg/mL TPCK‐treated trypsin. The sera were then serially diluted 1:2 across a clear, flat bottom 96‐well plate. The appropriate influenza virus was added at 100 TCID_50_ per well in infection medium with TPCK‐treated trypsin. A cell‐control and virus‐only control were included in each plate. Plates were incubated at 37°C, 5% CO_2_ for 1 h before adding MDCK cells (London strain) at 1.5 × 10^4^ cells/well in infection medium. The plates were then incubated at 37°C, 5% CO_2_ overnight. Influenza virus was detected in the cells in the same manner as the TCID_50_ assay. For diluted sera, percent inhibition was calculated in each well using the following formula: 1 − ((sample well OD—average OD of cell control)/(average OD of virus control—average OD of cell control)). Replicate wells were then averaged to determine percent inhibition at each serum dilution.

## 3 Results

### 3.1 MDCK Cells Were Adapted for Serum‐Free Suspension Growth

**FIGURE 1:**
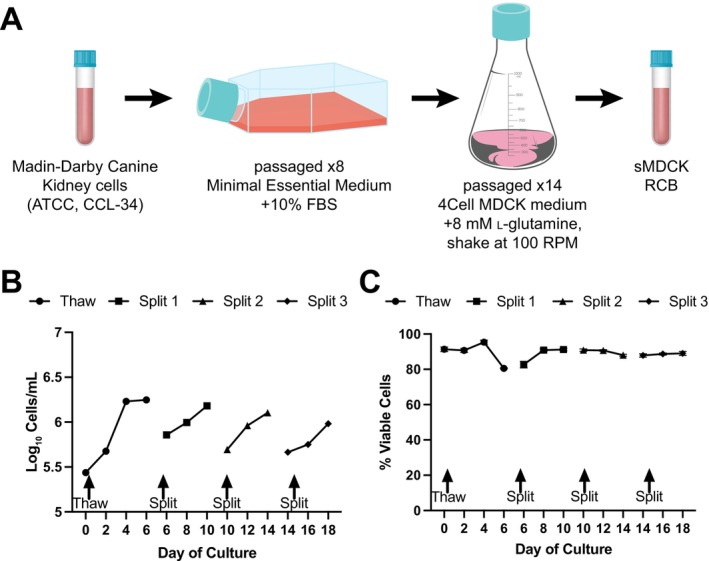
**Cell adaptation to serum‐free, suspension growth, and passaging of sMDCK cells.** (A) Schematic describing how off‐the‐shelf MDCK cells were adapted to serum‐free, suspension cell culture as described in the methods section. (B) sMDCK cell density during 18 days of routine culture, subculture days are noted on the graph. (C) sMDCK cell viability during 18 days of routine culture with subculture noted on the graph. (B and C) Data points represent the mean of three independent cultures.

Traditional adherent MDCK cells were adapted to serum‐free and suspension cell culture and underwent routine passage (Figure 1A). In our laboratory, the cost of the supplies and reagents used in sMDCK based viral propagation was 61.4% of the cost of adherent MDCK propagation. After adaptation, sMDCK viability was monitored and cells split regularly. A brief dip in viability was observed from day 4 to day 6 of cell culture, with percent viability dropping to approximately 80% (Figure 1B). This decrease in viability was likely the result of overconfluence, as sMDCK density remained stable from 4 to 6 days after thaw (Figure 1C). This indicates that there is an optimal cell density at which to split the sMDCKs, approximately 1.5 × 10^6^ cells/mL. Despite the initial viability drop, sMDCK cells quickly recovered and viability returned to normal levels after one passage. It is important to note that for optimum propagation, sMDCK cells need to be passed at least two times after thawing before infecting.

### 3.2 Cell Viability Drops Dramatically 48 h After Infection in sMDCK Viral Propagations

**FIGURE 2:**
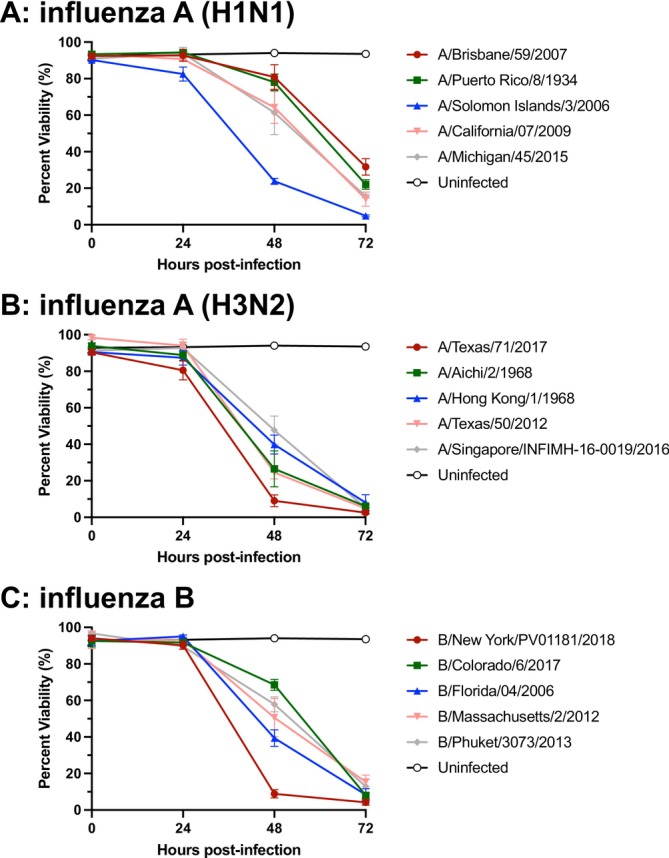
**Viability kinetics of sMDCK cells following influenza virus infection.** Viability was measured every 24 h using an automated cell counter and the trypan blue exclusion method. (A) Five H1N1 influenza strains, (B) five H3N2 influenza stains, and (C) five influenza B strains. For H1N1 and H3N2 strains, an MOI of 0.001 was used and influenza B strains were used at an MOI of 0.01. Data points represent the mean ± SD (*n* = 3).

To validate the sMDCK platform, influenza A and B strains were propagated at an MOI of 0.001 and 0.01, respectively. In each of the viral propagations, cell viability was greater than 90% at the time of infection, and this high viability persisted at the 24‐h mark, after which cell viability dropped off dramatically 2–3 days after infection. This cell death was not observed in the uninfected control, indicating reduced viability could be attributed to cytopathic effect as progeny virus were produced over the course of the propagation (Figure 2A–C). Therefore, cell viability measurements are a useful indicator of the status of infection and can be used for predicting the best time to harvest the newly produced virus.

### 3.3 sMDCK and Adherent Platforms Utilized to Grow 15 Influenza Strains Based on HAU Titer

**FIGURE 3:**
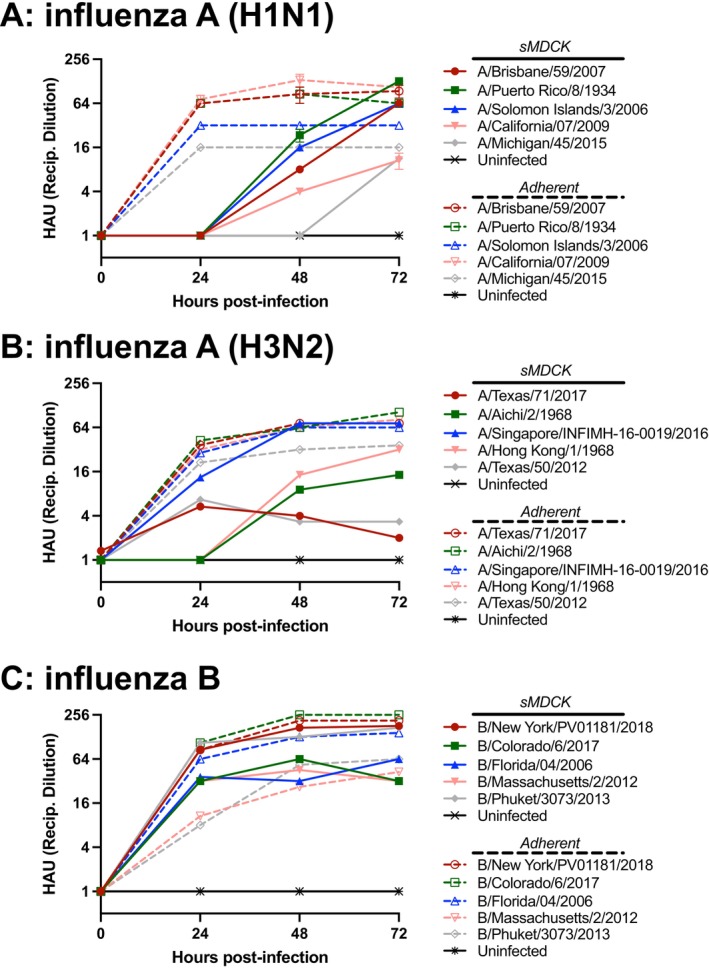
**Hemagglutination activity of influenza virus during 72 h of viral propagation using the sMDCK platform and adherent MDCK cells.** Samples were collected every 24 h, cryopreserved at −80°C, and HAU was determined using turkey RBCs. For each plot, solid lines are used to indicate virus grown using sMDCK cells and dashed lines are used for virus propagated on adherent MDCK cells. (A) H1N1 strains. (B) H3N2 strains. (C) Influenza B strains. Values less than the lower limit of detection (< 2 HAU) were set to 1 to facilitate visualization in each plot. Data points represent the mean ± SEM of three independent cultures for each virus at each time point.

To measure the viral titer at each time point, we used hemagglutination assays, a well‐accepted method of measuring influenza viral titer. For all H1N1 strains grown in sMDCK cells, with the exception of A/Michigan/45/2015, HAU titers were detectable after 48 h. The HAU titer for all of the H1N1 strains peaked at 72 h, indicating that the concentration of active hemagglutinin in supernatant was highest at this time point. We observed a wide range of HAU titers from 10.7 (A/California/07/2009) to 128 (A/Puerto Rico/8/1934) (Figure 3A). Comparatively, in adherent MDCK H1N1 propagations, all strains had a measurable HAU titer after 24 h.

For H3N2 influenza strains grown in sMDCK cells, A/Texas/71/2017 and A/Texas/50/2012 reached peak HAU titers 24 h after infection, and the other three strains (A/Aichi/2/1968, A/Hong Kong/1/1968, and A/Singapore/INFIMH‐16‐0019/2016) reached maximum HAU titers 72 h after infection (Figure 3B). Additionally, peak HAU titers ranged from 5.3 HAU (A/Texas/71/2017) to 72.8 HAU (A/Singapore/INFIMH‐16‐0019/2016). The A/Texas/71/2017 propagation produced detectable HAU titers at the time of infection. However, a single influenza reproductive cycle takes at least 5 h [24]. This positive HA value was most likely due to unbound or noninternalized virus from the initial infection step, rather than new virus produced. In contrast to the sMDCK propagations, H3N2 adherent MDCK propagations produced higher HAU titers, peaking between 2 and 3 days after infection.

For influenza B strains grown in the sMDCK platform, HAU were detected at 24 h after infection. The B/Colorado/6/2017 and B/Massachusetts/2/2012 propagations produced HAU values that peaked at 48 h, while the other three strains (B/New York/PV01181/2018, B/Florida/04/2006, and B/Phuket/3073/2013) produced maximal HAU titers 72 h after infection. The highest HAU titer each propagation yielded ranged from 45.3 HAU (B/Massachusetts/2/2012) to 181 HAU (B/New York/PV01181/2018) (Figure 3C). Similarly, influenza B virus produced in adherent MDCKs had measurable HAU titers after 24 h, with the maximum titer measured 2–3 days after infection.

Based on the WHO's standard [25], the minimum HAU titer for hemagglutination inhibition (HAI) assay input is 8 HAU. All of the propagated virus strains produced using the sMDCK and adherent MDCK platforms met this criterion, except for A/Texas/71/2017 and A/Texas/50/2012.

### 3.4 sMDCK Platform Used to Successfully Grow 15 Influenza Strains With Comparable Results in an Adherent Platform

**FIGURE 4:**
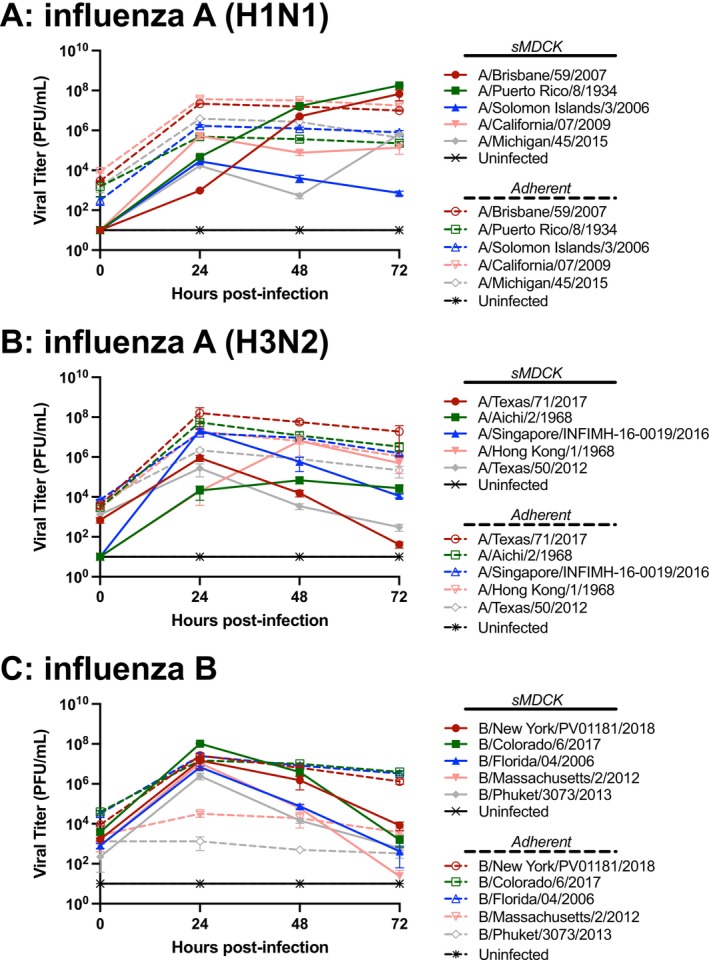
**Quantification of infectious virions produced using sMDCK cells and adherent MDCK cells.** Plaque assay was used to determine the concentration of infectious viral progeny during 72 h of propagation. In each plot, solid lines are used for data generated using sMDCK cells, and dashed lines are used for data from adherent MDCK cells. (A) H1N1 strains, (B) H3N2 strains, and (C) influenza B strains. Samples were collected and measured at 24‐h intervals after infection and held at −80°C until assayed. Values < 50 PFU/mL (LLOD) were set to 10 PFU/mL for inclusion in plot. Data points represent the mean ± SD; *n* = 3 for each strain at each time point.

In addition to hemagglutination units, infectious virions produced at each timepoint were quantified using a plaque assay. For H1N1 strains propagated in sMDCK cells, A/Solomon Islands/3/2006 and A/California/07/2009 reached a maximum titer 24 h after infection, and strains A/Brisbane/59/2007, A/Puerto Rico/8/1934, and A/Michigan/45/2015 reached a maximum titer 72 h after infection (Figure 4A). In sMDCK cells, maximum titers ranged from 2.83 × 10^4^ PFU/mL in A/Solomon Islands/3/2006 to 1.79 × 10^8^ PFU/mL in A/Puerto Rico/8/1934. Comparatively, in adherent MDCK cell propagations, PFU/mL titer peaked 24 h after infection, and titers ranged from 4.83 × 10^5^ PFU/mL in A/Puerto Rico/8/1934 to 3.67 × 10^7^ PFU/mL in A/California/7/2009.

Of the H3N2 strains grown in sMDCK cells, all of the viruses had a measurable PFU/mL titer 24 h after infection. For two of the viruses, A/Hong Kong/1/1968 and A/Aichi/2/1968, the PFU/mL titer continued to increase at the 48‐h time point before dropping at 72 h after infection (Figure 4B). Maximum titers ranged from 2.09 × 10^4^ PFU/mL in A/Aichi/2/1968 to 2.15 × 10^7^ PFU/mL in A/Singapore/INFIMH‐16‐0019/2016. In comparison for H3N2 strains grown in adherent MDCK cells, the peak titer was measured 24 h after infection. Except for A/Singapore/INFIMH‐16‐0019/2016, H3N2 strains generally grew to a higher PFU/mL titer in adherent MDCK cells with values ranging from 2.17 × 10^6^ PFU/mL in A/Texas/50/2012 to 1.60 × 10^8^ PFU/mL in A/Texas/71/2017.

For influenza B strains propagated in sMDCK cells, viral titer increased significantly at the 24‐h collection period, with each of the five influenza B viruses producing titers greater than 1 × 10^6^ PFU/mL. The PFU/mL titer dropped in each of the viruses at the 48‐h mark and again at the 72 h collection point (Figure 4C). Peak sMDCK PFU/mL titers ranged from 2.5 × 10^6^ PFU/mL in B/Phuket/3073/2013 to 1.04 × 10^8^ PFU/mL in B/Colorado/6/2017. A similar pattern in titer was observed for Influenza B virus grown in adherent MDCK cells, with infectious concentration peaking 24 h after infection and decreasing at each subsequent collection point. Titers ranged from 1.33 × 10^4^ PFU/mL in B/Phuket/3073/2013 to 2.58 × 10^7^ PFU/mL in B/New York/PV01181/2018.

Several propagations had detectable PFU/mL titers on the day of infection most likely due to noninternalized virus from the first step of the propagation. In general, we consider a virus stock useful if the titer is greater than 1 × 10^4^ PFU/mL for both in vitro and in vivo preclinical models. Except for the adherent propagation of B/Phuket/3073/2013, all strains produced in sMDCK and adherent MDCK propagations exceeded this standard.

### 3.5 Neutralization Assays With Known Antisera Confirmed the Maintenance of Antigenicity in sMDCK Progeny Virus

**FIGURE 5:**
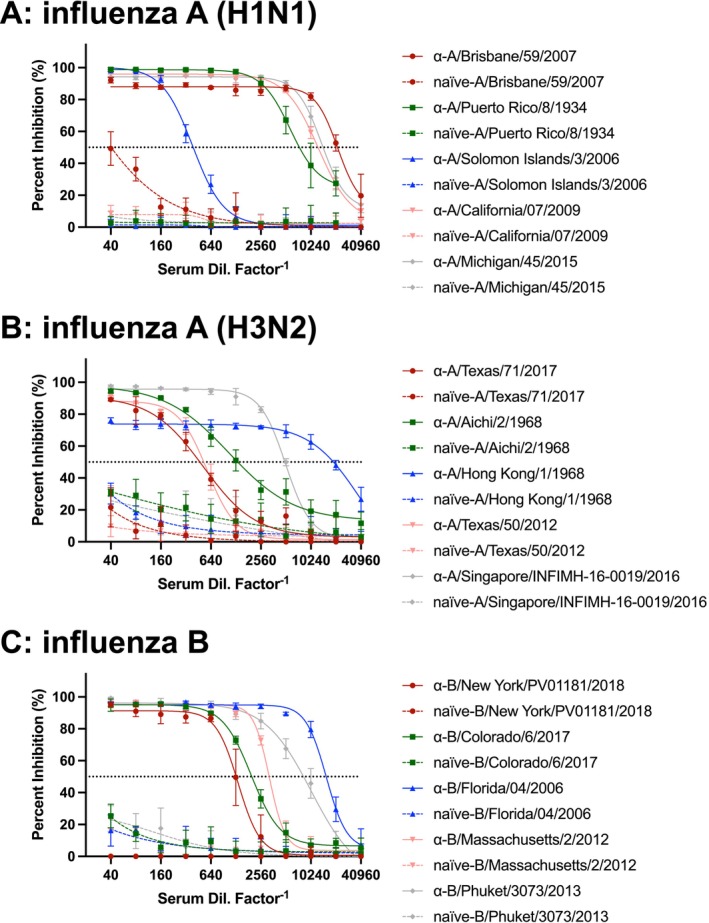
**Antigenic evaluation of influenza virus produced in sMDCK cells.** Each virus propagated using the sMDCK platform was titered by TCID_50_ assay, and 100 TCID_50_ of each virus was combined with respective antisera or nonreactive sera to assess antigenicity by microneutralization assay. Data from antisera are connected by interpolated solid lines, and data using nonreactive sera are marked with interpolated dashed lines. Antisera identity is listed in Table 2. Colorimetric data from the microneutralization assay were transformed to percent inhibition, and nonlinear regression analysis was used to generate neutralization curves for each virus strain and sera combination. Data in (A) are for H1N1 strains, (B) H3N2 strains, and (C) influenza B strains. Data points represent mean ± SD (*n* = 3–6 technical replicates).

Because preclinical vaccine research often relies on detecting neutralizing antibodies raised after a vaccination, virus inputs into neutralization assays must retain antigenicity similar to the circulating virus. To determine if viruses produced using the sMDCK platform maintained their antigenicity, we conducted neutralization studies using proven antisera against each strain of virus produced. Each H1N1 virus was combined with either strain specific antisera or sera from a naïve source (Figure 5A), and each strain was neutralized by specific antisera in a dose‐dependent fashion. Naïve antisera did not neutralize any H1N1 strain, with the exception of Brisbane/59/2007, where slight neutralization was observed in the least diluted conditions, but this inhibition did not exceed 50% inhibition. In the case of H3N2 strains, all viruses produced using the sMDCK platform were neutralized by specific antisera in a dose‐dependent fashion (Figure 5B), and no or very low neutralization was seen when treating the viruses with serum obtained from naïve sources. Similarly, all influenza B strains propagated in sMDCK cells were neutralized by strain‐specific antisera (Figure 5C), and no neutralization was observed using nonspecific antisera. Taken together, these findings indicated that viruses produced using sMDCK maintained their parental antigenicity, were susceptible to neutralization in the presence of specific antisera, and were not neutralized by naïve antisera.

## 4 Discussion

It is the goal of this work to demonstrate and share an alternative to traditional influenza propagation modalities using suspension cell‐based and serum‐free influenza propagation method that can be employed without the use of specialized equipment, reagents, or techniques. As described, sMDCK cells were generated by simultaneously adapting adherent MDCK cells to suspension and serum‐free culture, producing a robust population of cells that are relatively easy to passage. This sMDCK viral propagation approach has been shown to successfully propagate influenza viruses across many subtypes/lineages and isolation years, based upon our standards for a useful HAU and PFU/mL titer. After infection, cell viability and viral titers changed in predictable patterns. In all sMDCK viral propagations, we saw persistently high viability 24 h after infection, with that value dropping at the 48‐ and 72‐h timepoints. The majority of the sMDCK viral propagations produced a peak infectious titer, measured by plaque assay, at 24 or 48 h after infection before decreasing. This suggests that once cell death reaches a certain threshold in the sMDCK cells, further production of infectious virus slows, resulting in decreased infectious particles produced over time; this effect may be due to virus‐induced cell death or increased apoptosis in the sMDCK cell line as has been described [26]. In contrast, HAU titer peaked between 1 and 3 days after infection and then plateaued, consistent with previous findings that HAU titers can shift independently of infectious titer [27]. Additionally, microneutralization data suggests that virus produced using this platform maintains the correct antigenicity as confirmed with proven antisera. This finding indicates that viral products produced using the sMDCK platform will be useful in evaluating neutralizing efficacy of samples following vaccine or infection.

When comparing propagation titers between the suspension and adherent MDCK platforms, optimal PFU/mL and HAU titers varied on a strain‐by‐strain basis. Therefore, we offer this sMDCK platform not as a replacement for adherent propagation, but as an additional tool that offers a more scalable (we have produced virus in volumes ranging from 20 to 250 mL using sMDCK) and serum‐free alternative to traditional influenza propagation methods. This platform may also present a cost‐saving measure by eliminating the need for FBS, which is one of the most costly reagents in the adherent MDCK growth medium that is required leading up to propagation. Additional optimization of MOI and harvest time may be warranted based on the strain to be propagated. It has been the case in our lab that the described protocol works, but strain‐specific optimizations can be made to ensure optimal results. While the sMDCK platform does not necessarily produce higher titers than adherent MDCK, it is widely successful in propagating a wide variety of influenza strains to comparable titers. Furthermore, regular passage of adherent MDCK cells requires FBS, an expensive product with costs exceeding $500 per 500 mL. Through the elimination of this costly reagent, the sMDCK platform offers a potential cost‐saving measure, making it more accessible to laboratories with limited resources.

As noted in the introduction, several groups have created serum‐free suspension MDCK cell lines for propagation of influenza [1, 9, 11, 15, 16, 17, 18, 19, 20]. These lines have been used to propagate typical laboratory influenza strains (e.g., A/Puerto Rico/8/1934 (H1N1), A/California/07/2009 (H1N1)) as well as vaccine and avian strains of interest. The diversity of methods in those papers make it difficult to perform a direct comparison, but the data presented here indicates that the sMDCK cell line and the described protocol has the potential to be useful for virus propagation both for vaccine development and for influenza research.

In addition to the utility of the sMDCK platform described, the approach detailed in this report could be applied to adapt genetically modified adherent MDCK cell lines to serum‐free suspension culture. For example, adherent MDCK cells have been “humanized” to express high levels of human‐like influenza receptors [6, 15, 28]. Such strains have proved more successful in maximizing growth of H3N2 influenza strains. In our comparison of traditional adherent MDCK and suspension MDCK platforms, H3N2 strains were the only subtype in which adherent cells produced consistently higher titers. Adaptation of these genetically modified MDCK cell lines to serum‐free suspension using this protocol could be used to potentially generate high‐titer H3N2 influenza stocks at a dramatically reduced cost.

The sMDCK platform has been used consistently in our lab to produce large volumes of influenza virus for use in various research models. We have distributed several hundreds of vials to collaborators, replaced older stocks, generated new stocks, and used these viruses as input material to test 1000s of samples in neutralization assays. These virus stocks have been used to supply large research programs such as the Collaborative Influenza Vaccine Innovation Centers (CIVICs) group to ensure that investigators are able to conduct their research using the same high‐quality virus input, aiding in assay concordance between institutions and laboratories. The sMDCK cells used in this protocol will be made publicly available through biorepositories such as BEI Resources (NR‐59612) to ensure more research groups can grow their own influenza stocks, using a unified method, to conduct vital surveillance and preclinical research in the affected settings. The protocol described in this work could increase the number of researchers capable of working toward the next generation of influenza countermeasures and vaccines.

## 5 Conclusions

Our goal was to demonstrate a platform to propagate influenza virus that can be conducted and adapted in a wide variety of laboratories. The processes described in this work resulted in the production of influenza virus stocks that were of comparable titer to traditional methods. Importantly, this protocol can be adapted for a given strain to maximize progeny virus recovery. Egg‐based and adherent cell‐based propagation remain extremely useful, and we see our work as adding more tools that can be used to combat the ever‐present threat of influenza infections.

## Author Contributions

**Jessica B. Huskey:** conceptualization, investigation, writing‐original draft, writing‐review and editing, data curation, formal analysis.**Michelle L. Rock:** investigation, writing‐review and editing, data curation, formal analysis, visualization. **Pooja V. Chaudhary:** investigation, data curation, writing‐review and editing. **Emily C. Hill:** investigation, data curation, writing‐review and editing. **Madeline E. Hoover:** investigation, data curation, writing‐review and editing. **Nicole M. Rideout:** investigation, data curation, writing‐review and editing. **Kamerin D. Dean:** investigation, data curation, writing‐review and editing. **Thomas Scott Alderman:** project administration, writing‐review and editing. **Phong Ho:** resources, investigation, writing‐review and editing. **M. Anthony Moody:** project administration, funding acquisition, supervision, writing‐review and editing. **Gregory D. Sempowski:** project administration, funding acquisition, supervision, writing‐review and editing. **Thomas H. Oguin III:** Conceptualization, supervision, investigation, writing‐review and editing.

## Funding

This work was supported by the National Institutes of Health (UC6‐AI058607, G20‐AI167200, UC7‐AI180254), the National Institute of Allergy and Infectious Diseases (75N93019C00050), and the Research Triangle Institute.

## Conflicts of Interest

The authors declare no conflicts of interest.

## Data Availability

The data that support the findings of this study are available from the corresponding author upon reasonable request.
